# Medical 3D printing for vascular interventions and surgical oncology: a primer for the 2016 radiological society of North America (RSNA) hands-on course in 3D printing

**DOI:** 10.1186/s41205-016-0008-6

**Published:** 2016-12-01

**Authors:** Leonid Chepelev, Taryn Hodgdon, Ashish Gupta, Aili Wang, Carlos Torres, Satheesh Krishna, Ekin Akyuz, Dimitrios Mitsouras, Adnan Sheikh

**Affiliations:** 1 The Ottawa Hospital Research Institute and the Department of Radiology, University of Ottawa, 501 Smyth Road, Box 232, Ottawa, Ontario K1H 8L6 Canada; 2 Faculty of Medicine, University of Ottawa, Ottawa, Canada; 3 Department of Radiology, Applied Imaging Science Lab, Brigham and Women’s Hospital, Boston, MA USA

**Keywords:** 3D Printing, Aneurysm repair, Cancer, Segmentation, Computer-aided design, Orthopedic Surgery, Implant, Surgical Guide, Radiological Society of North America, Precision Medicine

## Abstract

Medical 3D printing holds the potential of transforming personalized medicine by enabling the fabrication of patient-specific implants, reimagining prostheses, developing surgical guides to expedite and transform surgical interventions, and enabling a growing multitude of specialized applications. In order to realize this tremendous potential in frontline medicine, an understanding of the basic principles of 3D printing by the medical professionals is required. This primer underlines the basic approaches and tools in 3D printing, starting from patient anatomy acquired through cross-sectional imaging, in this case Computed Tomography (CT). We describe the basic principles using the relatively simple task of separation of the relevant anatomy to guide aneurysm repair. This is followed by exploration of more advanced techniques in the creation of patient-specific surgical guides and prostheses for a patient with extensive pleomorphic sarcoma using Computer Aided Design (CAD) software.

**Electronic supplementary material:**

The online version of this article (doi:10.1186/s41205-016-0008-6) contains supplementary material, which is available to authorized users.

## Introduction

In the short interval since the publication of our initial practical medical 3D printing guide for the 2015 annual RSNA meeting [1], the published literature in this domain has undergone exponential growth. The number of peer-reviewed journal publications has nearly doubled, ever expanding the breadth and scope of the applications of 3D printing in medicine. It is evident that 3D printing is poised to play an important role in transforming the practice of medicine, with applications ranging from fabrication of simple tools to complex tissues and, eventually, organs. Development of familiarity with 3D printing may therefore be of considerable interest to a wide range of medical professionals.

The term “3D printing” has evolved to become synonymous with the terms “rapid prototyping” and “additive manufacturing” within the medical domain, and refers to the process of fabrication of 3D objects through sequential deposition and fusion of matter in a layer-by-layer fashion [2]. A wide range of printing technologies are available to enable the fabrication of 3D models in a range of materials, including plastics, metal alloys, ceramics, and numerous biological substrates supporting living cells. While a more detailed examination of these technologies is covered at length elsewhere, it must be noted that this diversity enables a tremendous range of applications at numerous levels of cost, accuracy, durability, build time, and biocompatibility. Medical 3D printing is therefore nearly universally accessible and applicable.

The process of 3D printing starts with the generation of a printable model in a process that is not entirely dissimilar to typical 3D reconstructions. Typically, cross-sectional imaging is used as an initial step in model creation. Computed Tomography (CT), by virtue of reflecting changes in a single parameter (attenuation), is the preferred modality for this purpose. The acquisition of images specifically for the purposes of 3D printing requires minimizing the size of voxels within the reconstructed images while optimizing contrast administration for adequate visualization of the relevant anatomy.

Once appropriate images are acquired, segmentation, or isolation of the relevant anatomy is undertaken using methodology that is akin to 3D visualization methods available in most radiology departments. The process of segmentation involves creating a set of criteria for the voxels to satisfy in order to be included in a model. These criteria may include connectivity to a seed point, attenuation coefficient within a specific range, or presence within specific manually defined geometric boundaries. Such selection criteria may be set manually or specified automatically as part of applying a segmentation algorithm.

While 3D visualization typically ends at selecting and displaying a set of segmented voxels, further additional model manipulations are required to enable 3D printing. Fundamentally, the collection of voxels occupying a specific set of Cartesian coordinates within a region of interest (ROI) needs to be transformed into a 3D object in a process referred to as *tessellation*. Tessellation is widely used in computer graphics to approximate shapes using a set of triangles. The more triangles are used, the more refined a shape becomes. Unfortunately, transformation into a 3D object does not ensure model printability or stability. For instance, it may be necessary for the model to be further smoothed, non-printable parts may be removed or mathematically adjusted, and vulnerable areas may be reinforced using a range of automated algorithms.

Further manipulations using Computer-Aided Design (CAD) software may be necessary to enable the development of patient-specific instrumentation, implants, or reconstructions. Given the ubiquity of such tasks, this course will focus not only on segmentation of patient anatomy, but also the *de novo* creation of personalized medical models.

The principles of 3D printing in medicine are best appreciated through practical hands-on experiences covering a broad range of applications. Therefore, this guide will provide the foundational knowledge to broadly cover the segmentation of relevant anatomy on CT-derived Digital Imaging and Communications in Medicine (DICOM) images followed by 3D printable model creation and concluded by patient-specific reconstructions and surgical guide design.

The United States Food and Drug Administration (FDA) classifies medical 3D printing software into *design manipulation software* that enables medical device design and modification and *build preparation software* that enables the conversion of the digital design into a file format that is 3D printable, or Standard Tessellation Language, or STL file in this case [3]. To illustrate the use of the former, we shall apply Mimics inPrint and 3-matic Medical software (Materialise, Leuven, Belgium) while the latter will be represented by Polyjet Studio (Stratasys Ltd., MN, USA).

The Mimics inPrint software facilitates the processing of 2D image data acquired from axial imaging (CT, MRI) in order to create 3D printable models. While the vast majority of functionality is present in this accessible package, certain higher-order operations, including various segmentation algorithms present in more advanced packages such as Mimics (Materialise, Leuven, Belgium), are omitted for simplicity and accessibility.

3-matic Medical is a CAD package tailored for design of medically relevant models. This software enables the manipulation of patient-derived 3D models as well as creation and redesign of entirely new models in the context of patient anatomy. The application of this package in anatomical reconstruction and medical device creation will be explored in this work.

Finally, Polyjet studio enables the preparation of the 3D models represented as a set of connected triangles in an STL file format for printing on a commercial 3D printer. This software enables the exploration of factors such as part placement, material costs, build times, and material selection in order to optimize the 3D printing process. While the Polyjet studio is dedicated specifically to Polyjet printers capable of producing multi-colored prints, the general interface and approach are fairly representative of the experience in setting up a 3D printing task.

In the first case, we shall examine two common scenarios where 3D printing holds the potential of improving patient care: pre-procedure planning for vascular interventions with an example of iliac artery aneurysm repair and creation of surgical tools for neoplasm resection on a patient with a large pleomorphic sarcoma.

Iliac artery aneurysms are mostly seen in association with aortic aneurysms. Isolated iliac artery aneurysms are rare, involving <0.1 % of population [4]. Most common etiology is atherosclerosis, while less common causes include connective tissue disorders, trauma, dissection, or infection. The most commonly involved site is the common iliac artery, followed by the internal iliac artery [5]. Aneurysms of external iliac artery are relatively rare.

Although most patients are asymptomatic, some present with symptoms due to mass effect on adjacent ureters, nerves, or veins. Rarely, aneurysmal rupture will lead to an acute presentation. Asymptomatic aneurysms are treated when they enlarge beyond 3–3.5 cm in diameter or if they become symptomatic [6–8]. Although treatment can be performed by open surgery or by an endovascular approach, the latter is presently favored due to its safety and lower complication rates, except in patients with compressive symptoms [9, 10].

Depending on the location and extent of the aneurysm, endovascular treatment involves deployment of stent graft with or without internal iliac artery embolization or surgical bypass. Since internal iliac embolization can lead to complications including buttock claudication, bowel ischemia, sexual dysfunction, and tissue necrosis, branch iliac artery grafts are also used to preserve flow, especially while treating bilateral aneurysms.

An initial CT angiogram is performed prior to the procedure to define the anatomy and select the appropriate device. Pertinent information sought includes assessment of access (common femoral artery) for its diameter (large enough to accommodate delivery system), presence of plaques or calcifications, which can hinder access. Iliac artery diameters at landing zones (typically 15–20 mm length) are measured in true short axis using multiplanar reconstructions to select the size of the stent graft (typically oversized by 10- 15 % or as per manufacturer’s recommendation). Iliac vessel tortuosity is also assessed as it can pose a challenge during the procedure.

A 3D printed model can therefore be quite beneficial in preoperative planning and teaching of aneurysm repairs. The aneurysm model produced using this process may be used to demonstrate the mechanism of stent deployment in endovascular aneurysm repair. Using this case as a guide, learners will become familiar with segmentation techniques used routinely in 3D printing to expose relevant anatomy, as well as extrusion techniques necessary to reconstruct the anatomy of any contrast-opacified structure, including vasculature, heart, or hollow viscus.

The second case demonstrates the design and surgical planning involved in the creation of a prosthetic hip implant for a patient with an extensive lytic lesion within the left iliac crest secondary to a soft tissue sarcoma. This patient was diagnosed with undifferentiated pleomorphic sarcoma (giant cell subtype).

Pleomorphic sarcoma is the most common type of high grade soft tissue sarcoma in the adults [11, 12]. It often presents as an aggressive, large, high grade sarcoma of the extremity. Prognostic risk factors include tumor size, depth, and proximal location. This soft tissue sarcoma is thought to be derived from a primitive mesenchymal cell capable of differentiating into histiocytes, fibroblasts, myofibroblasts, and osteoclasts. The etiology of the tumor remains unknown. Prior radiation therapy is likely a risk factor. The most common clinical presentation is an enlarging painless soft-tissue mass in the thigh, typically 5–10 cm in diameter. The majority of tumors are intramuscular.

When it arises within the pelvis, detection may be limited due to its deep location, and the patient may only seek medical attention when the tumor is quite large. Although it may metastasize to lungs or local lymph nodes, this neoplasm typically expands and recurs regionally to produce a lesion which may visually appear to be well circumscribed but may actually have extensive soft tissue invasion [11]. The treatment of choice is wide local excision with adjuvant radio- and chemotherapy, for a cumulative 5 year survival on the order of 50–60 % of all high-grade pleomorphic sarcomas [13–15].

**Fig. 1:**
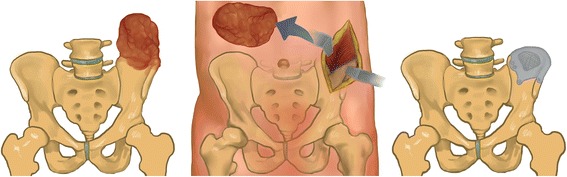
Simplified overview of pleomorphic sarcoma supported in this work. After identifying the extent of the neoplasm (left, red), it is resected with wide margins. The skeletal defect is filled using a patient-specific implant (right)

The patient presented here (patient TCGA-QQ-A5V2, The Cancer Imaging Archive [16]) has wide involvement of the iliac crest and the wide excision would be planned using a custom cutting guide, followed by reconstruction of the excised bone fragment using a customized titanium implant (Fig. 1). The design of the implant will be based on the disease-free hemipelvis.

This case will primarily focus on CAD software (3-matic) to introduce the learners to the patient-specific surgical planning and reconstruction principles that may then be adapted to a wide range of cases, ranging from craniotomy implants to surgical planning with custom guides for osteotomies involving all bones of the appendicular and axial skeleton.

**Fig. 2:**
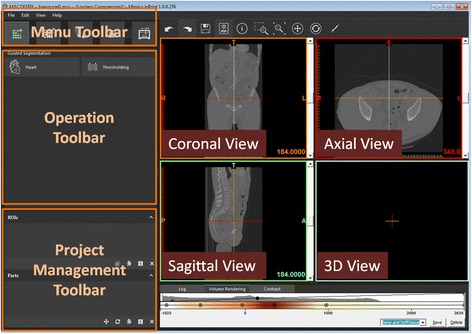
Overview of the Mimics inPrint project screen

Prior to beginning the learning modules, the reader is directed to an overview of Mimics inPrint software and controls (Fig. 2, Table 1).

**Table 1:** Mimics inPrint keyboard and mouse shortcuts

Shortcut	Action
Scroll Wheel Drag (2D View) OR Shift right click + drag	Pan: Move the mouse while keeping the center button pressed
Right Click + Drag (2D View)	Zoom: click and drag the right mouse button to zoom in and out.
Arrow Up/Scroll Up (2D View)	Go to next slice
Arrow Down/Scroll Down (2D View)	Go to previous slice
Page Up (2D View)	Skip 10 slices upward
Page Down (2D View)	Skip 10 slices downward
Hover mouse over a view, then SPACE	Change chosen image to/from full screen.
Backspace	Switch between two window states
CTRL + Z (Everywhere)	Undo the previous action.
CTRL + Right Click + Drag (2D View)	Adjusts contrast window in images
Right Click + Drag (3D View)	Rotate a 3D shape.
CTRL + Right Click + Drag (3D View)	Zoom into or out of 3D shape.
Shift + Right Click + Drag (3D View)	Pan the 3D shape around a scene.

### Patient 1: endovascular aneurysm repair simulation

#### Task A: segmentation

WHAT YOU ARE DOING: Segmenting the aorta and iliac vessels as well as the bones. In ***segmentation***, specific voxels satisfying specific attenuation criteria are selected to create a region of interest. You will isolate the vessels and bones in this step.

WHY YOU ARE DOING IT: To identify the voxels for conversion into a 3D model.

HOW TO DO IT: We will be using a widely available tool, ***Thresholding***, to select the desired voxels based on attenuation within a specified Hounsfield Unit (HU) range. This step will create a ***Region of Interest (ROI)***, an intermediate model that requires further manipulations to be printed. A list of the ROIs you have created appears in the first pane of the Project Management Toolbar (bottom left).

We shall begin by importing the images into our project from a DICOM folder. The DICOM data can be obtained from the OsiriX DICOM image library [17]. To do this, select the **File** menu, then **New From Disk**, followed by selecting the **DICOM Data** folder, pressing the **Next** button in the dialog that appears, and finally **Convert**. This will load all the DICOM images into the workspace.

**Fig. 3:**
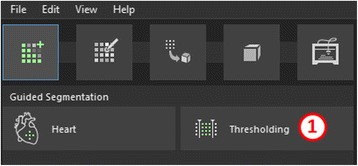
Thresholding function (1) within the Guided Segmentation menu

In order to segment the aorta and the iliac vessels, select Thresholding (Fig. 3).

**Fig. 4:**
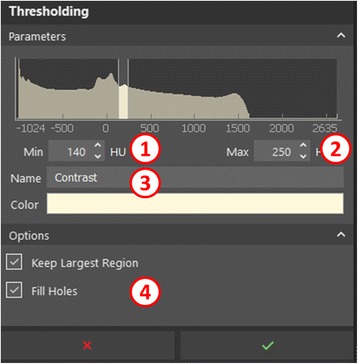
Settings for minimal (1) and maximal (2) attenuation thresholds, naming the ROI (3), and options (4)

This will bring you to a menu that allows you to provide the HU range for the anatomy of interest. Since we would like to select contrast-enhanced vessels while avoiding bones and soft tissues, we will define a fairly narrow range of values, from 140 to 250 (Fig. 4). Rename the ROI to ***Contrast***, and select in the options to keep only the largest region and fill holes in the ROI. This will provide us with the smoothest contiguous model possible.

**Fig. 5:**
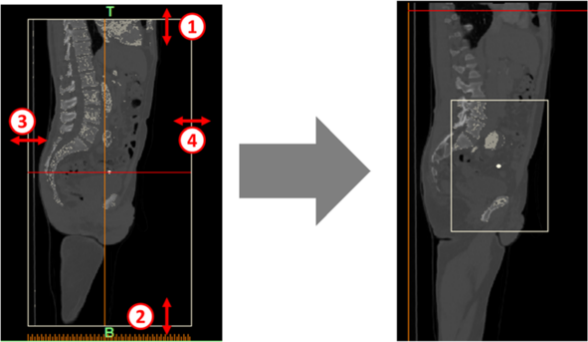
Adjustments for the bounding box for the segmentation in the sagittal midline (1–4)

After the settings for segmentation are in order, adjust the segmentation box by moving its outlines on the sagittal view to only include the relevant pelvis and lower abdomen. Specifically, move the superior boundary to the L3-L4 disk level, posterior to S1-S2 level, and inferior to just below the inferior pubic rami (Fig. 5). Anterior adjustments are not necessary.

**Fig. 6:**
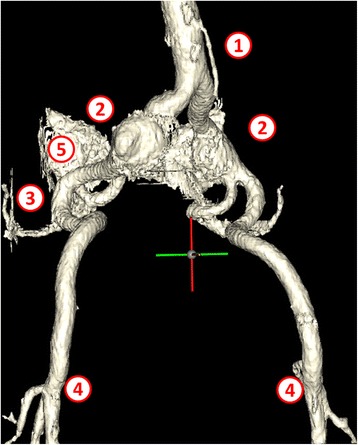
Intermediate segmentation ROI demonstrates the mesenteric artery (1), large bilateral common iliac artery aneurysms (2), superior gluteal arteries (3), as well as the origin of the deep femoral and femoral circumflex arteries bilaterally (4). Note the remnants of the sacrum (5) which need to be separated from the vascular structures

Click the checkmark in the thresholding setup. After calculations complete, the ROI will be revealed in the 3D View (Fig. 6). The inferior abdominal aorta and its branches are quite well resolved in the resultant ROI, though you will notice that the segmentation is incomplete as remnants of the sacrum require separation from the vasculature.

**Fig. 7:**
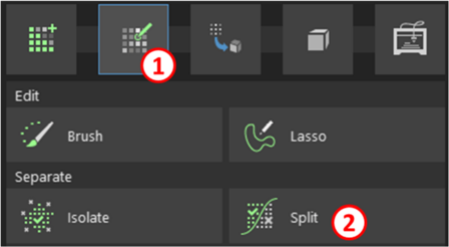
The Edit ROI menu (1) and the Split tool (2)

In order to remove the remnants of the sacrum from our ROI, we will use the **Split** tool in the **Edit ROI** menu (Fig. 7). This tool allows us to define a **Foreground**, or all structures to be included in the ROI and a **Background**, or all structures to exclude.

***Segmentation***

Segmentation is a task that simply creates a set of criteria for voxels to fulfil in order to be included in a model. The first set is created by the ***thresholding*** function – a range of HU values are set to select the relevant voxels. When we decided to “***keep the largest region***” after thresholding, we selected the largest set of contiguous voxels. With the split function, we are providing a number of seed points for the software to automatically extend in order to create two dissimilar contiguous regions, separating the anatomy we wish to see in our model from the background.

**Fig. 8:**
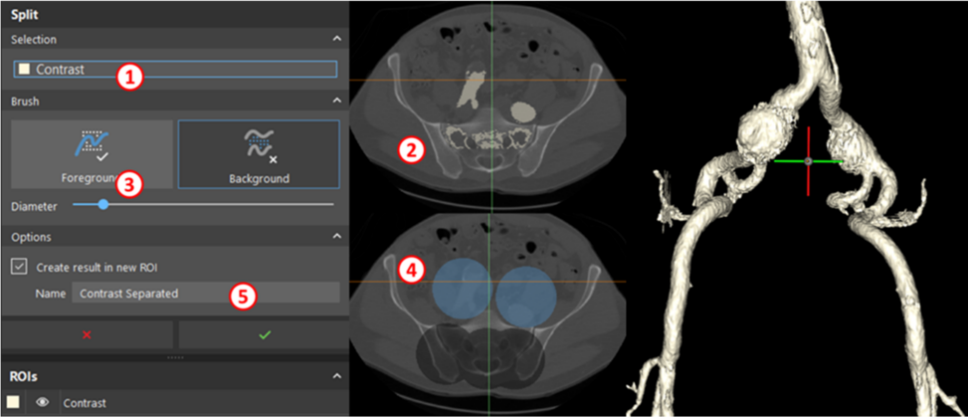
Selecting the Contrast ROI (1), moving to the bifurcation of the right common iliac artery (2) and selecting the foreground (3) and background (4) structures to create a new separated ROI (5), right

Note: *To expand the axial image window to the full screen, simply press the spacebar while hovering over it with your mouse.*

In order to do this, first ensure that the Contrast ROI is selected, choose an axial image that shows both, the vascular structure ROI to be included and the skeletal structures to be excluded at the level of the right common iliac artery bifurcation (axial image annotated 397.5 in left lower corner) and paint the foreground (ROI volume to remain in the model) as well as the background (ROI volume to remove) on this axial image (Fig. 8). Once this is complete, ensure you **create result in new ROI**, name the ROI “Contrast Separated” (5), and press the green checkmark. If you have done this correctly, a new ROI comprised only of vascular structures will appear, as shown.

Thus far, we have *segmented* the contrast-opacified arteries within the patient’s lower abdomen and pelvis. This has created an *ROI* or *mask*, which is simply a collection of voxels that satisfy a set of criteria, which in this case constitute 1) 140-250HU density range, 2) membership within the largest contiguous collection of voxels satisfying density criteria, and 3) algorithmic separation of ROI based on user input. This is quite similar to segmentation that is typically performed in 3D rendering on a daily basis. Note that this collection of voxels is not yet a 3D printable model. In order to create a printable model, we need to convert this collection of voxels into an STL model and verify that this STL model is printable.

#### Task B: creation of prinable STL models

***What are STL models?***

STL models define a 3D geometry as a collection of triangles that collectively describe a shape. As the number of triangles used increases, the approximation of reality by the model improves, but the model becomes more computationally expensive to create and manipulate. The mere act of describing a model as a set of triangles does not ensure printability, however. Further processing is required to ensure that the shapes that are represented in the STL model are *manifold* shapes and can exist as non-abstract geometrical constructs. While this is outside the scope of this tutorial, non-manifold shapes include entities such as planes, lines, and points, which have no thickness, cannot exist in the real world, and will likely lead to errors in printing.

**Fig. 9:**
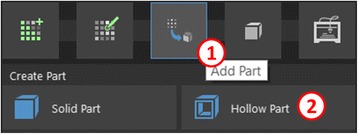
Add part menu (1) and the Hollow Part function (2)

To create an STL model of the vessels, we will simply use the *Add Part* function to create a *Hollow Part* (Fig. 9). Creating a hollow part as opposed to a solid part allows us to model vessels by creating a 1.5 mm wall around the intraluminal contrast we have just segmented.

**Fig. 10:**
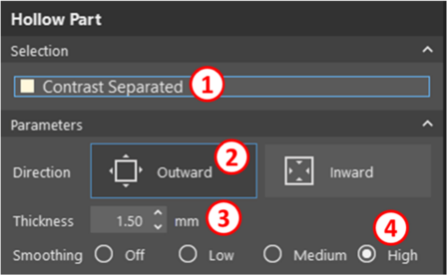
Selecting the ROI (1) and setting the parameters (2–4) for hollow part creation

In the **Hollow Part** dialog that appears, we will ensure that the ROI we have created is selected (Fig. 10), then select an outward direction to create walls *outside* the contrast ROI, set wall thickness to an arbitrary value of 1.5 mm, smoothing to High, and click the green checkmark.

**Fig. 11:**
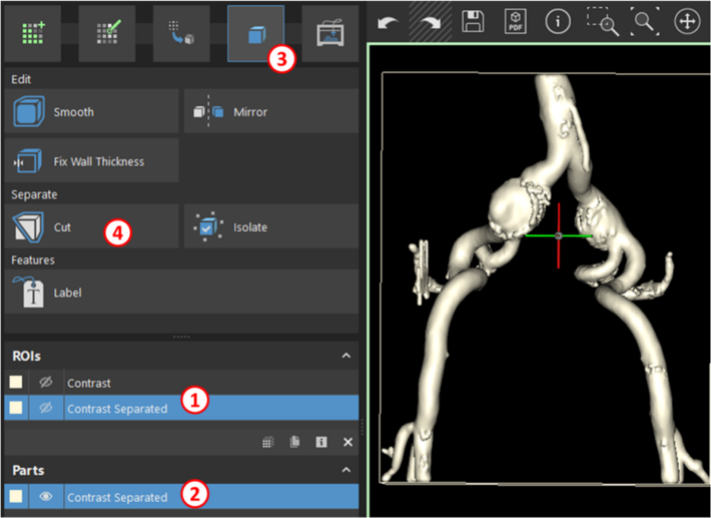
The ROI is hidden (1) and a part is created (2) which will then be edited (3) using the Cut function (4)

This will create an STL model, also known as a *Part* within the inPrint software. The ROI will be hidden at the end of the operation, as denoted by the crossed out eye within the list of ROIs, and a new part named *Contrast Separated* will appear (Fig. 11). The software automatically opens the *Edit Part* section.

**Fig. 12:**
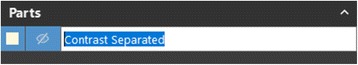
Renaming an ROI

To reflect the transformation of the model, rename part we just created from “Contrast Separated” to “Arteries” by single-clicking the text label of the part that was just created, typing its new name, and pressing ***Enter*** on the keyboard (Fig. 12).

After inspecting the model, you will notice that a uniform wall was made around the intraluminal contrast ROI. Since we would like to simulate stent placement, it is necessary to open the vessels by cutting off the closed ends. To do this, select the **Cut** function.

**Fig. 13:**
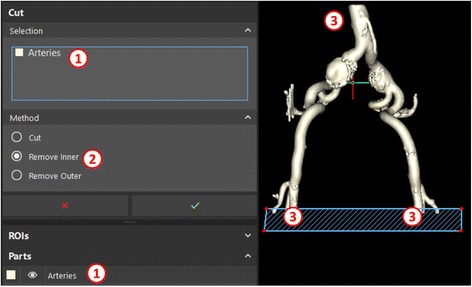
Selecting the Arteries part (1) and removing the inner part (2) of rectangular selections at the proximal and distal ends of the part (3)

We will first reorient the model so that we are looking at it face-on to allow us to perform a smoother cut in one plane. For this, use the **View** menu, and navigate to **3D Viewports > Views > Front**. Then, in the **Cut** dialog (Fig. 12), ensure that the *Arteries* part is selected, select the option to remove the inner part of the selection you highlight, and draw a rectangle around the three vessel ends, pressing the green checkmark for each individual cut to carry out the procedure (Fig. 13).

**Fig. 14:**
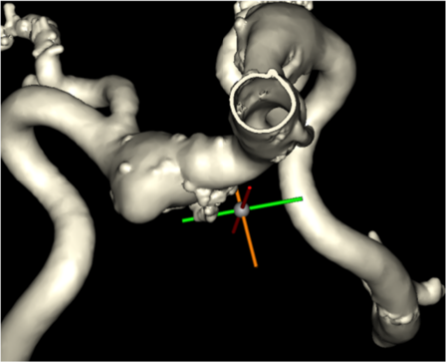
Completed model of the vasculature including the iliac aneurysms

With the completion of this task, you should be able to explore the model and look inside the simulated vessels (Fig. 14). The vascular model is now complete. Detailed instructions regarding model printing are provided in Additional file 1.

### Patient 2: soft tissue sarcoma excision and personalized implant design

For this patient with extensive soft tissue sarcoma invading into the osseous structures of the pelvis, we shall first need to design a patient-specific prosthetic implant. To do this, we would require to mirror and duplicate the healthy hemipelvis exactly at the point of excision. In order to allow for precise excision that spares healthy tissue, we would also need to create cutting guides to direct the wide excision of this neoplasm in a manner that allows subsequent placement of the custom implant flush with the excision site while optimizing the resection volume.

The images for this project may be obtained from the Cancer Imaging Archive [16] by searching for the patient TCGA-QQ-A5V2 and retrieving corresponding CT images (no direct link is available). The preparation of these CT images for segmentation is identical to that in Task A for Patient 1.

#### Task A: segmentation

**Fig. 15:**
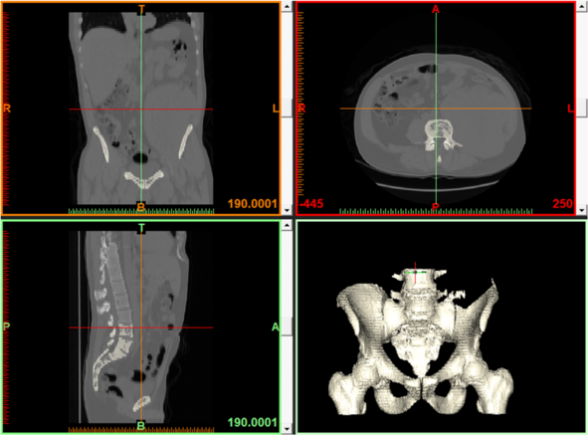
Result of bone segmentation. Note the missing left iliac crest (lower left image)

To begin operative planning, we will first need to visualize the involvement of the skeletal structures of the pelvis with the large soft tissue sarcoma by thresholding between 250HU and 1520HU and limiting the bounding box at the L3-L4 intervertebral disk proximally. The steps for this are identical to those in Task A for Patient 1. Limiting the bounding box in any other dimension is not necessary. If you have successfully completed these steps, you will be able to obtain a model similar to Fig. 15. Name your ROI **Skeletal**.

#### Task B: isolation of relevant pathology

**Fig. 16:**
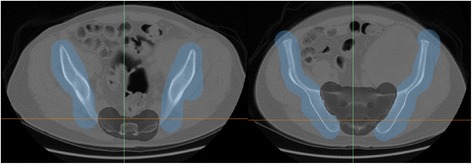
Two isolated levels demonstrating the foreground (blue) and background (black) selection

In this step, our goal is to isolate only the iliac bones. We will take advantage of the Mimics inPrint **Split** function as we did earlier, within the **Edit ROI** menu. With this tool, ensure that the **Skeletal** ROI appears in the Selection list as before. The **Foreground** brush will again be used to paint all structures that should be preserved, while the **Background** brush will be used to indicate structures to reject (Fig. 16).

The axial view is best suited for selection of the background (rejected) and foreground (desirable) features in this case. The sacrum will be the background, while the remainder of the bones of the pelvis will be the foreground. You need not paint all of the axial images – two well-placed images will typically suffice. If your selection results in more remnant fragments than necessary, more axial images may be annotated. After you are satisfied with your result, press the green checkmark in the **Split** dialog.

**Fig. 17:**
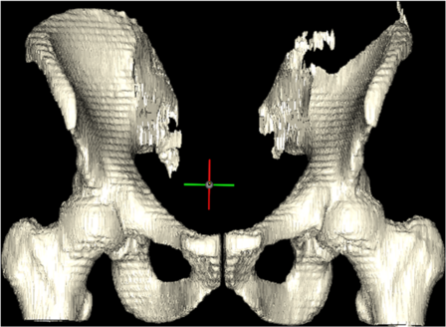
Result of applying the Split function to the pelvis, separating the sacrum from the iliac bones

Upon the completion of the operation, you shall see that the separation product in the ROI list and the 3D view (Fig. 17). In the resultant model, the femurs are still in place—these will be useful for alignment at a later time.

**Fig. 18:**
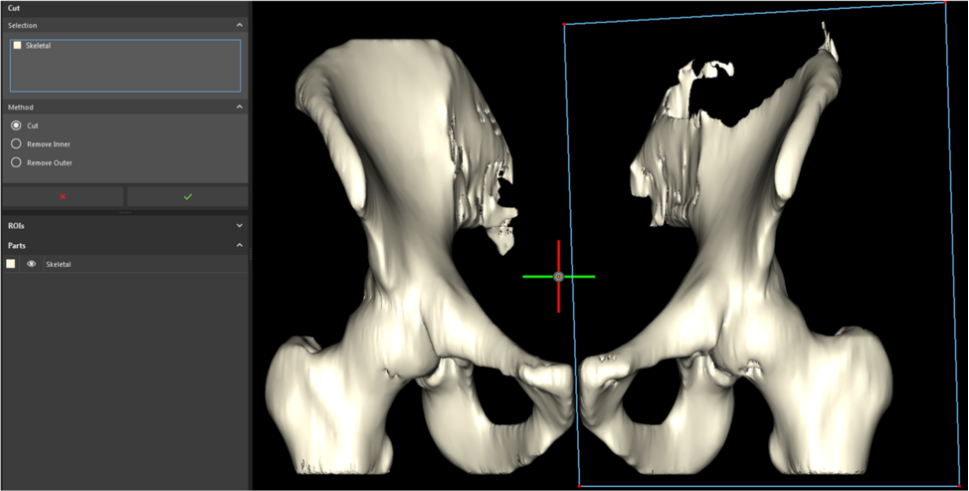
Separation of the left and right hemipelvis along the symphysis pubis

Transitioning to an STL model, we shall now create a part using the **Add Part** menu, this time generating a **Solid Part** with **High** smoothing, as shown earlier, making sure that the **Skeletal** ROI appears in the list. This will generate a smoothed STL model ready for further processing (Fig. 18).

#### Task C: mirroring the healthy hemipelvis

***Mirroring***

Mirroring is a basic approach used in the reconstruction of patient anatomy in cases of severe pathologic unilateral deformities. Since humans exhibit plane symmetry through the midline sagittal plane, the non-pathologic side can be *mirrored* and computationally overlapped with the diseased side to provide rapid patient-specific reconstruction. This technique is widespread, from craniotomy plate creation to mandibular reconstruction, for example. In this work, we shall use the healthy hemipelvis as a template for the hemipelvis with the sarcoma. For this, we will need to first separate the two halves of the pelvis, and then reflect and overlap the healthy hemipelvis with the diseased one.

After the part has been created, the software will display the **Edit Part** menu. Here, we shall select to **Cut** the part into two. Before doing this, however, let us align the view to best prepare the cut. Go to **View** in the menu toolbar, and then select **3D Viewports > Views > Front**. Once this is set up, ensure that the **Skeletal** part is in the selection menu, that the **Method** is set to **Cut**, and draw a rectangle separating the two halves of the pelvis, as shown. Press the green checkmark button once this is complete.

**Fig. 19:**
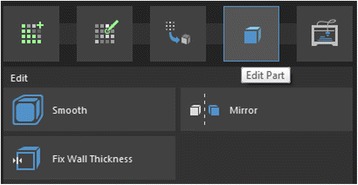
The Edit Part menu and the Mirror function

This action will create two parts, **Skeletal-inner** and **Skeletal-outer**, which we will rename these parts **Left** and **Right** respectively (if you have set up the cut as in Fig. 18), to reflect the anatomy. We shall first mirror the healthy right half to use as a template for the reconstructive implant. To do this, return to the **Edit Part** menu and select the **Mirror** function (Fig. 19).

**Fig. 20:**
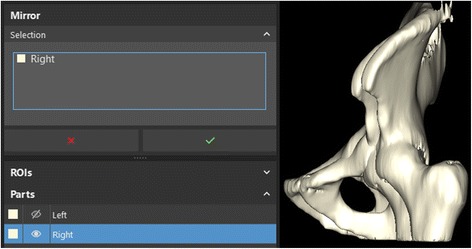
Mirror menu selection and the result upon mirroring the right hemipelvis

Select the healthy hemipelvis (**Right**) here and execute the mirroring by pressing the green checkmark (Fig. 20). After this process is complete, delete the **Right** model by selecting it in the Parts list and pressing **Delete** on the keyboard.

You will notice that a **Right_Mirrored** part has now been created. Visualizing the two parts together as above, you will notice that their alignment is not complete, limiting implant creation. Computer Aided Design (CAD) software, **3-matic** will be necessary to complete this task. To transfer our work, simply save the project from the **File** menu.

#### Task D: computer-aided implant design with 3-matic

**Fig. 21:**
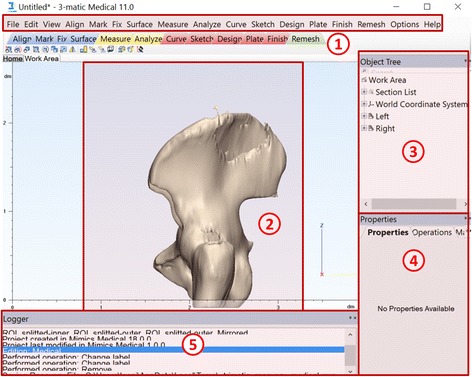
Main window overview of 3-matic. Featured are the menu toolbar (1), 3D view (2), Object tree (3), Properties (4), and the Logger (5)

The 3-matic CAD software contains powerful tools for editing any STL file in preparation for printing as well as for creation of new 3D printable parts. We will demonstrate several of these tools to familiarize you with the capabilities of this software. To retrieve the project in 3-matic, simply import the project saved earlier by pressing **File > Import Part** and locating the project in the dialogue that appears. This will open the two overlapping hemipelvis models created earlier, as shown (Fig. 21).

You will notice that the 3-matic window has a **Menu Bar** where its numerous functions can be found, a **3D view** to visualize the selected models, **Object Tree** where the manipulated models appear, **Operations Tab** which allows us to set parameters for various functions, and the **Logger** to display pertinent status updates (Fig. 21).

**Fig. 22:**
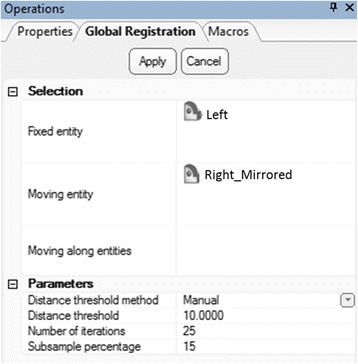
Global Registration alignment operation setup

The Mimics inPrint work stopped when we discovered imperfections in the alignment of the two models. Fortunately, 3-matic has a built-in function to align two models: select **Align > Global Registration** on the menu bar. In the **Operations Tab** for the Global Registration function, select the **Fixed entity** to be the **Left** hemipelvis, the **Moving entity** to be the mirrored **Right** hemipelvis, and set the parameters as shown, with distance threshold of 10 and number of iterations at 25. Press apply (Fig. 22).

**Fig. 23:**
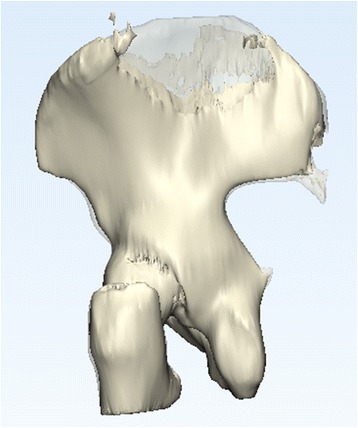
Overlapping hemipelvis models

Once the process completes, you will see excellent alignment of the two models. Right-click the **Right** model on the object tree and select **View > Transparency > High** to better visualize the overlap of the two parts (Fig. 23).

**Fig. 24:**
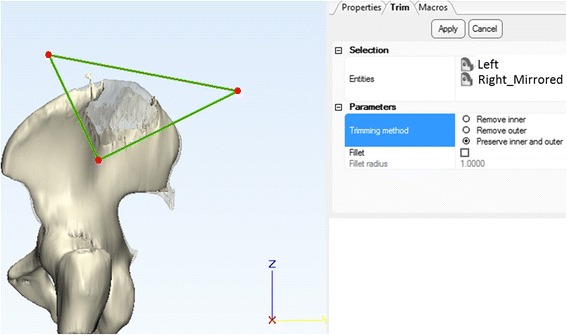
Setting up the trim operation to plan surgical excision

Now that the two models are overlapping, we shall plan the surgical cut. First, set the view to the right (**View > Default Views > Right)**—this will allow us to standardize our cut while ensuring that the cutting planes are orthogonal to the ZY plane. Select the **Trim** function from the **Finish** menu and set it up to **preserve inner and outer** fragments and to operate on both parts of the pelvis, as shown. Press **Apply** when ready (Fig. 24).

**Fig. 25:**
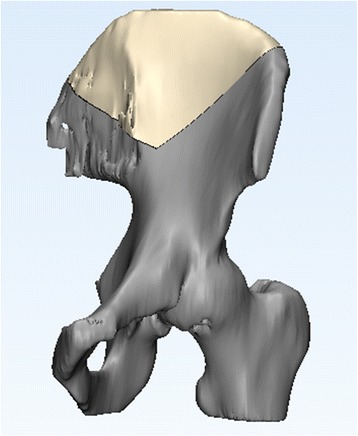
The healthy portion of the right hemipelvis (black) and the implant (beige)

This will create four fragments from the previous two. Deleting the two unnecessary fragments—the resected tumor and the unused part of the healthy hemipelvis by selecting them on the Object Tree and pressing **Delete** on the keyboard, we can appreciate the excellent alignment of the newly designed custom implant (beige) in relation to the diseased hemipelvis (black) (Fig. 25). If this alignment is unsatisfactory in your case, fine adjustments may be carried out by scaling, rotating, and translating the implant model as needed within 3-matic.

#### Task E: computer-aided cutting guide design

Let us now take a moment to rename all model parts for clarity. Select the smaller part of the **Right_Mirrored** model and rename it to ***Implant***. To do this, simply single-click the name of this object in the Object Tree so that it changes to an editable field, type in the new name and press **Enter**. This is the reconstructed, patient-specific implant that will be printed in titanium and implanted after the resection. Because we have completed our manipulations with it, we shall now hide it by right-clicking it in the Object Tree and selecting **Hide**. Likewise, rename the healthy part of the left hemipelvis as **Left**. The object tree should therefore contain only two parts: **Left** and **Implant**.

We shall now create a surgical guide for more precise excision of the diseased portion of the left hemipelvis, using the patient’s own hip geometry. To do this, we shall first return to the Right view (**View > Default Views > Right**). We shall then **Wrap**, **Cut**, **Translate**, and finally carry out a **Boolean Subtraction** on the left hemipelvis to create the beginning of a simple surgical template to guide the excision.

***Wrapping Operation***

The wrapping operation fills all holes and defects inside our model and creates a solitary watertight surface that would be easily amenable to 3D printing. In this operation, the model is checked to ensure that non non-manifold geometry is present in it. This is important because all objects within the real world are manifold: it is impossible to manufacture a perfect plane (single fold) of 0 thickness, or two shapes that touch in a single, mathematically discrete dot – these are abstract concepts. The wrapping operation ensures that the geometry of our model is not only watertight, devoid of internal cavities, and smooth, but also that it can exist in the real world!

**Fig. 26:**
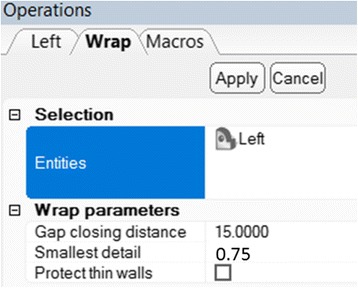
Setup of the wrapping operation

Select **Fix > Wrap** from the menu bar and set up the Wrap function as shown. In several seconds, this operation will remove any holes within the model that are below 15 mm wide (gap closing distance), while smoothing all imperfections that are smaller than 0.75 mm (Fig. 26).

This operation will result in a new wrapped model, **Left_wrapped**. Hide the **Left** model and duplicate the **Left_wrapped** model by right-clicking it and selecting **Duplicate**. We will now operate on this duplicated model (**Left_wrapped_duplicate**) to create a surgical guide that adheres to patient anatomy.

Hide the **Left_wrapped** model and select the **Trim** function from the **Finish** menu. Reset the view of your model to **Right** (**View > Default Views > Right**) so that we are again creating cuts within the ZY plane. The reason for this will become apparent in a moment.

***Landmarks and the Surgical Guide: Notes from the OR***

In our case, the orthopaedic surgeon consulted for this case would like to position the patient in the **right lateral decubitus** position. This exposes the lateral border of the left pelvis for resection. The surgeon would then proceed to resect the diseased soft tissue and **dissect** the healthy musculature from the **smallest possible portion** of the lateral aspect of the iliac bone. In doing so, the surgeon would attempt to preserve the abdominal wall if possible, while **minimizing** the amount of muscle tissue detached from the lateral aspect of the left pelvic bone while skeletonizing it. The surgeon would then prefer to use the **anterior superior iliac spine** as the first and most obvious landmark for surgical guide placement, followed by the **posterior superior iliac spine** as the second point of alignment. If the surface of the surgical guide conforms to the lateral surface of the iliac bone while leaving a **1 mm margin of error** for anticipated minimal imperfections while skeletonizing it, the surgical guide is anticipated to fit adequately. Once aligned to all landmarks, the **inscriptions on the implant** are again verified to ensure appropriate placement. The guide is then secured in place by **positioning pins** placed within **appropriate holes** placed on the implant at the design stage, and the guided osteotomy can begin.

**Fig. 27:**
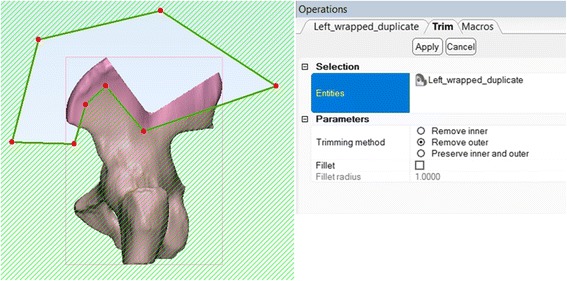
Intermediate step in creating the surgical guide, trimming

Since we would like to include the anterior superior iliac spine (ASIS) and the posterior superior iliac spine (PSIS) as landmarks within our surgical guide while minimizing the amount of soft tissue resected to accommodate our guide, we will have to **trim** our model accordingly. Select the **Trim** function from the **Finish** menu and draw the boundaries (Fig. 27). Ensure that the wrapped model duplicate appears in the Entities list and select ‘**Remove outer’** as the trimming method.

**Fig. 28:**
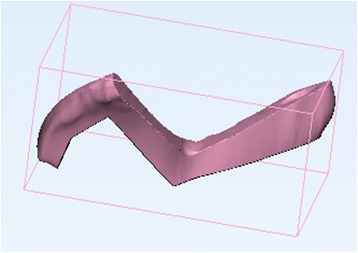
Result of trimming the left hemipelvis to sculpt a surgical guide

This will produce a fragment that now resembles a surgical guide (Fig. 28).

We shall now use this fragment to carry out a **Boolean subtraction**, which will create a fragment that adheres to the lateral surface of the iliac bone and mimics the patient’s anatomy closely, assisting the surgeon in its placement. Therefore, we will simply **move** this fragment lateral to the iliac bone and then **remove its overlap** with the iliac bone, leaving a 1 mm margin to accommodate any imperfections of surgical resection.

**Fig. 29:**
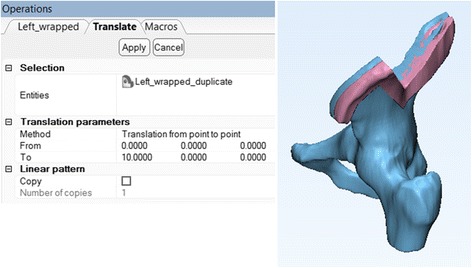
Translating the surgical guide precursor and translation result

First, use the **Translate** function from the **Align** menu (Fig. 29). Ensure that the translation **Method** is set to ‘Translation from point to point’, the **From** point is set at the origin (0,0,0) while the **To** point is set 10 mm in the positive direction along the X axis (10,0,0). Because we only cut our models orthogonally to the ZY plane earlier, this move is also orthogonal to the ZY plane and allows us to create a guide with a lateral surface parallel to that of the iliac bone and a guiding surface that is parallel to the direction of the osteotomy saw. Effectively, this ensures the best alignment while allowing the osteotomy saw to rest against the guide, assuring the excision is performed as planned. Press **Apply** and **Show** the **Left_wrapped** model (right click on it in the Object Tree and select **Show**). If performed correctly, you should see the two overlapping models.

**Fig. 30:**
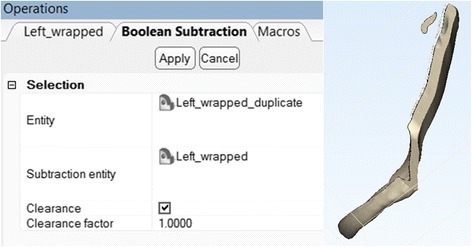
Boolean subtraction and its result

Now, we are able to carry out a **Boolean Subtraction** as shown (**Design > Boolean Subtraction**). To take into account imperfections of skeletonization of the iliac bone, ensure that **Clearance** is selected and set it to 1 mm as shown (Fig. 30). This operation will remove the two models involved and will create instead a model called **Subtraction result – 001**. Rename it now to **Cutting Guide** from the Object tree.

**Fig. 31:**
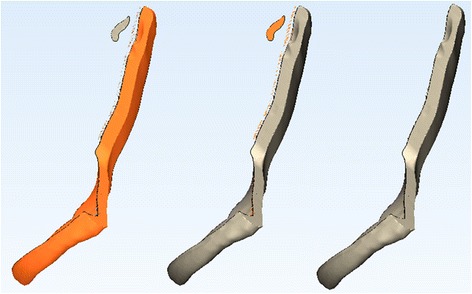
Intermediate steps in clearing excess shell fragments

You will notice that this model contains a number of imperfections along its edge. To remove them, simply mark the fragment you would like to keep using the **Mark > Shell** operation, which will select the shell (collection of contiguous connected triangles) that corresponds to the surgical guide itself. Now, **Mark > Invert** the selection to select all the smaller shells to discard. Press the **DEL** key on the keyboard to delete these fragments (Fig. 31).

**Fig. 32:**
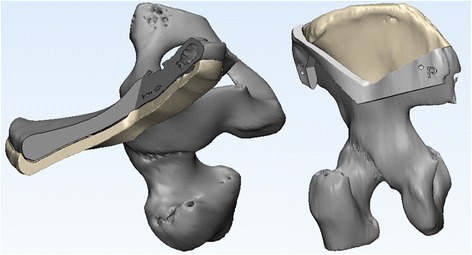
Result of guide creation (left) and the final model visualization after surgical guide annotation and pin position placement (right)

To visualize the results, **Show** the **Left** model from the **Object Tree** and explore the alignment. Once you are satisfied, visualize the implant by **Showing** it from the Object Tree as well. The guide and the implant are now complete, and with minimal further perfection with external labels and holes for positioning pins discussed in Additional file 2, a surgical plan is complete and personalised medical instrumentation and implant can now be printed (Fig. 32).

These models can now be exported as STL files (**File > Export > STL**) and printed. A further overview of 3D model printing is discussed in Additional file 1.

## Conclusion

As the applications of 3D printing in medicine continue to expand, familiarity with the basic operations and tasks involved in this process becomes increasingly important. By the virtue of their existing functions, some radiologists may very well subspecialize to emerge as physicians responsible for integrating the input from other specialists to design procedure guides and generate medical implants in the future. In this work, our goal has been to familiarize the medical professionals with this nascent field and start the dialogue in this revolutionary development in personalized medicine that has the potential of transforming the practice of medicine entirely.

## Additional files

Additional file 1: Printing a 3D Model with Polyjet Studio. (DOCX 1049 kb)

Additional file 2: Creating Holes and Labeling. (DOCX 193 kb)

## Abbreviations

3D: Three dimensional
ASIS: Anterior superior iliac spine
CAD: Computer-aided design
CT: Computed tomography
DICOM: Digital imaging and communications in medicine
FDA: United States Food and Drug Administration
HU: Hounsfield units
MRI: Magnetic resonance imaging
ROI: Region of interest
RSNA: Radiological Society of North America
STL: Standard Tessellation Language
