# Supplementary material for: Medical 3D printing for vascular interventions and surgical oncology: a primer for the 2016 radiological society of North America (RSNA) hands-on course in 3D printing
Source: 3D Print Med. 2016 Dec 1;2:5. doi: 10.1186/s41205-016-0008-6 (PMC6036767; doi:10.1186/s41205-016-0008-6)
Supplement: Supplementary file 1 — Printing a 3D Model with Polyjet Studio. (DOCX 1049 kb) [file 41205_2016_8_MOESM1_ESM.docx]

Appendix B: Printing a 3D Model with Polyjet Studio

We will demonstrate a typical printing job in Polyjet Studio using the Hemipelvis and implant models we’ve created

**WHAT YOU ARE DOING:** Using a new software platform called ***Polyjet Studio*** to place the Hemipelvis and implant on the “build tray”, the platform on which the model will be built. After visualizing the guide and implant from different perspectives, we will assign the material and color and “send” the job to the 3D printer.

**WHY YOU ARE DOING IT**: In ***Mimics*** and ***3-matic***, we have generated the STL file. ***Polyjet Studio*** is a software package associated with the 3D printer and it is used to organize print jobs, select the materials, and execute printing.

**HOW TO DO IT**: The STL file will be imported into the new software package. Next, the file will be rendered exactly as it will be 3D printed on the build tray. The orientation can then be adjusted, and the material and color of choice selected. Build time and material usage will also be estimated.

We will now switch software packages and will simulate the printing of the Hemipelvis and implant model using ***Polyjet Studio***.

The main screen of ***Polyjet Studio*** is shown below.


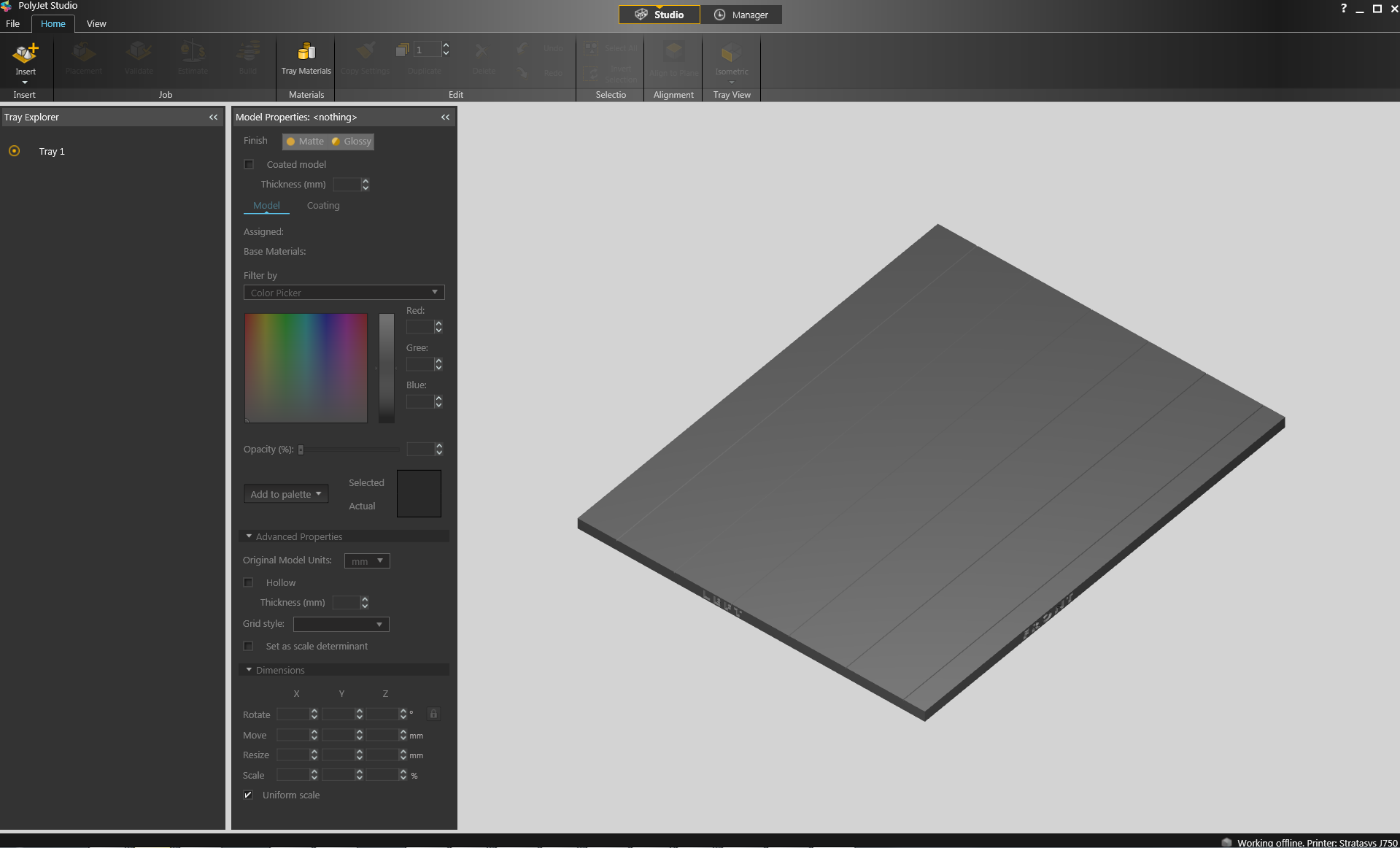


1. To import the Hemipelvis and implant guide STL file, click the upper left menu icon **Insert->Insert Model**, and select both STL files from the dialog box that opens by left mouse clicking.

*Hint: make sure that you press and hold CTRL on your keyboard before left clicking on the STL files to select multiple files at once.*

Our models will be automatically imported onto the software representation of the build tray of the printer.


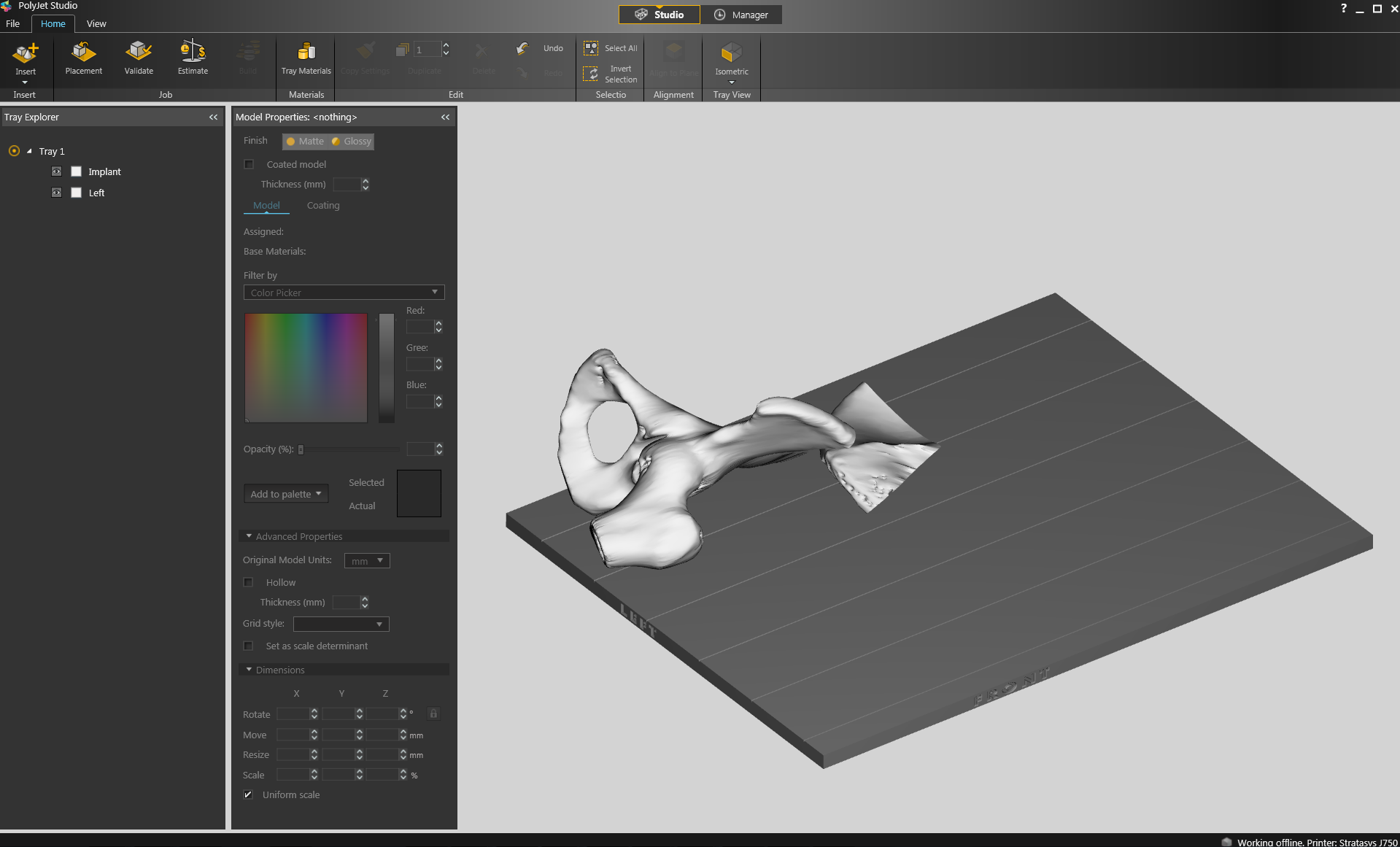


The orientation of the Hemipelvis and implant is optimized so that the support material and time needed for the 3D printing job can be minimized. For example, it would be possible, but far less efficient, to print the Hemipelvis standing “upright” rather than “lying down”.

Note: to import the models connected as an assembly, we may want to use the **Insert->Assembly** option. The models will be printed together, but we will still be able to assign different materials to each.


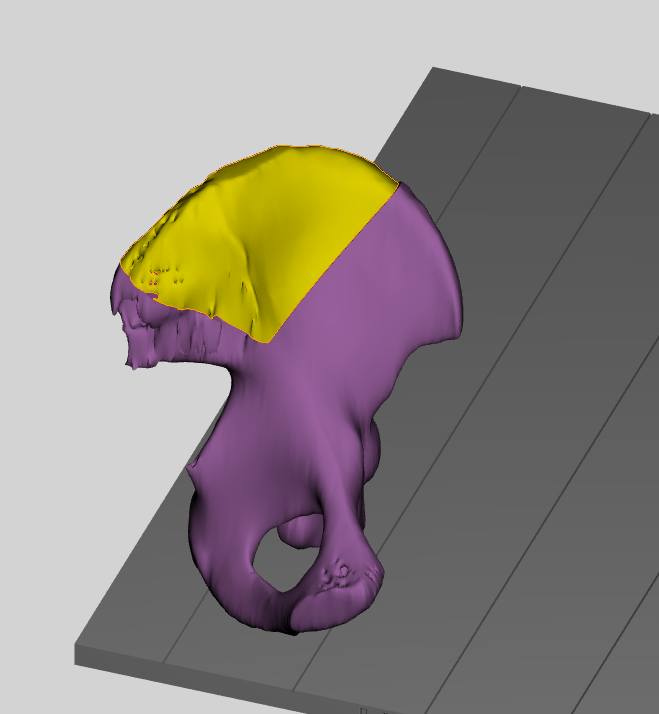


1. Visualizing the model on the build tray. The default view of the entire build tray of the 3D printer is in a single isometric view. By using the mouse wheel, you may zoom to visualize one model on the screen


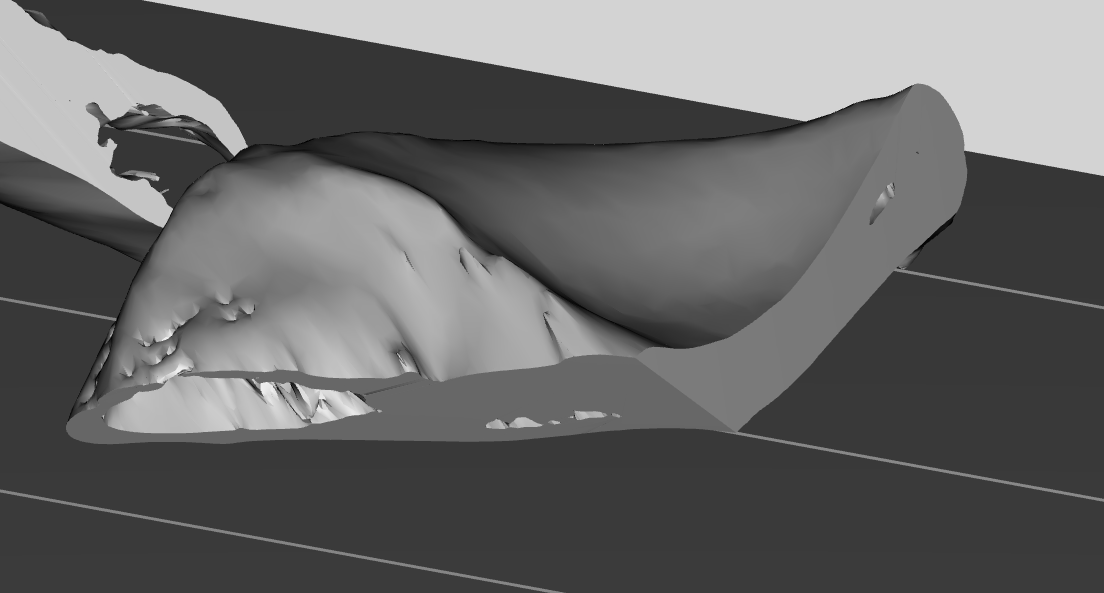


In practice, with several objects on the build tray, it is important to be able to change the viewing orientation. This can be done by clicking the **Tray View** icon
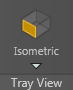
, and navigating the pull-down menu to different views such as **Top, Left, Right, Back** and various Isometric views. This series of commands will change the viewing orientation og the tray with the result as shown in figure below.


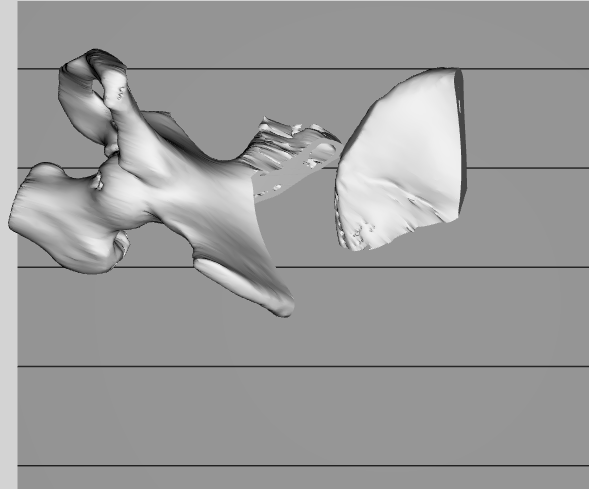


For certain printing jobs we might be required to modify the orientation of the model to minimize printing time and material consumption. To perform this, first we will choose the “Left” model from the tray explorer


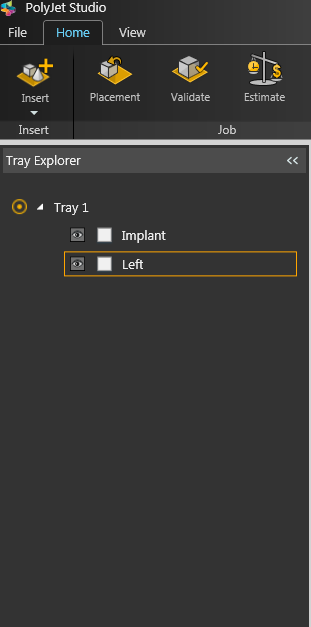


We will choose the “Back” view from the **tray view** icon.


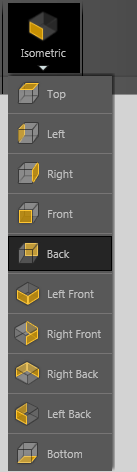


This will enable us to see the models height so we may rotate it


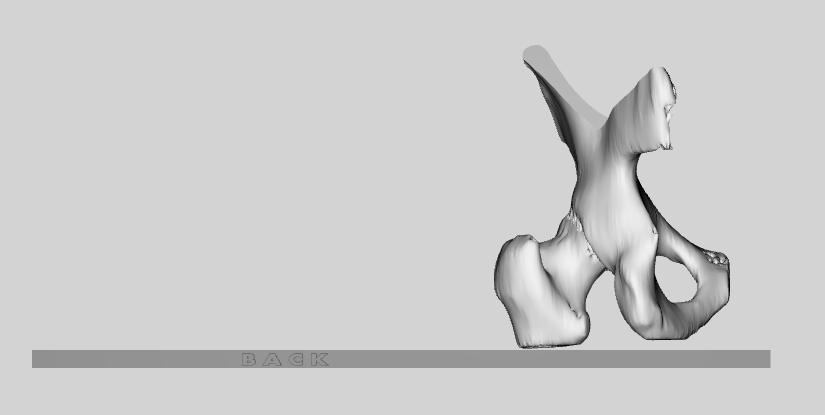


To rotate the model, we can either click the arrows or input a number, in degrees, that we would like to rotate the model by. Experiment with the different axis.


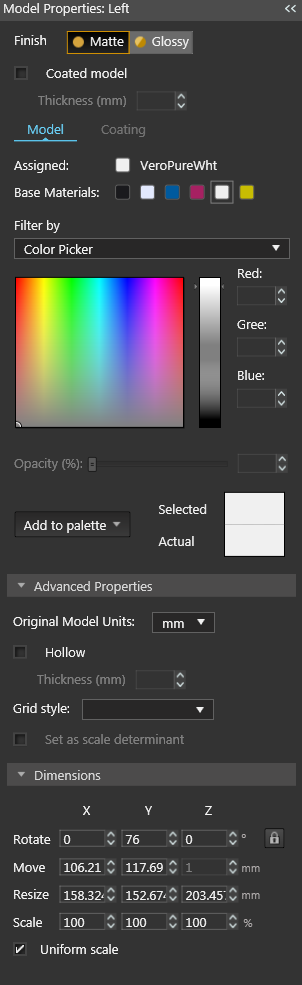


Another way to minimize the time/support consumption is to use **automatic placement.** To perform this, click the “Placement” icon on the “job” tab of the menu.
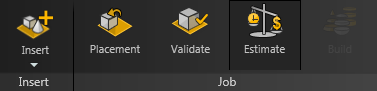


1. Next, we will assign the material of choice in order to build the model and start the 3D printing job. . We will change the material setting of the Hemiplevis to light gray and the Implant to VeroClear, our transparent material. To do this, select the first model (“left”) from the **tray explorer**.


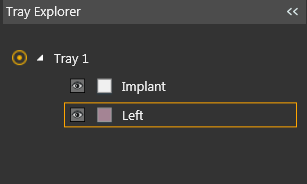


To change the color we can choose our desired color, from the **color picker** section of model properties window


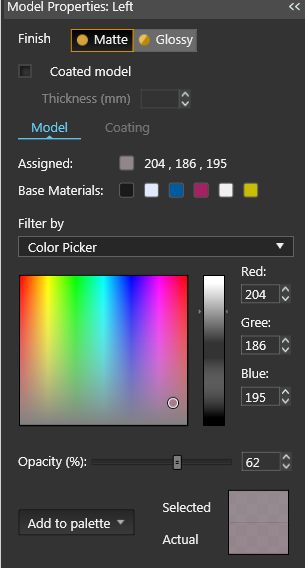


Our model will change color, reflecting the full color capabilities of the J750 platforms.

To assign a transparent VeroClear material to our implant model, we will choose it from the tray explorer


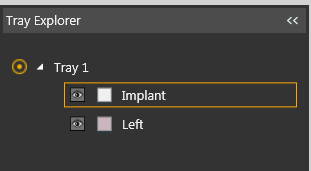


On the **model properties** window, we will choose VeroClear from the **base material** section


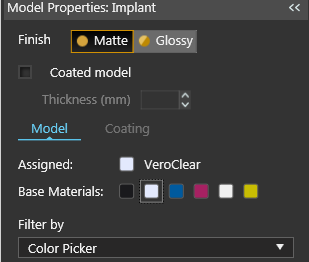


1. Estimating the build time and material usage before sending to the 3D Printer. Simply click the **Estimate** icon
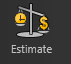
 and ***Polyjet Studio*** will prepare a material usage estimate and build time. This icon can be found in the upper left of our screen and selecting it will open up the **Production** **Estimate** window. Due to the lack of a server, ***Polyjet Studio*** will not be actively connected to a printer. Build times and consumption of material can vary depending on the printer. You can now close the **Production** **Estimate** window by selecting **OK**.


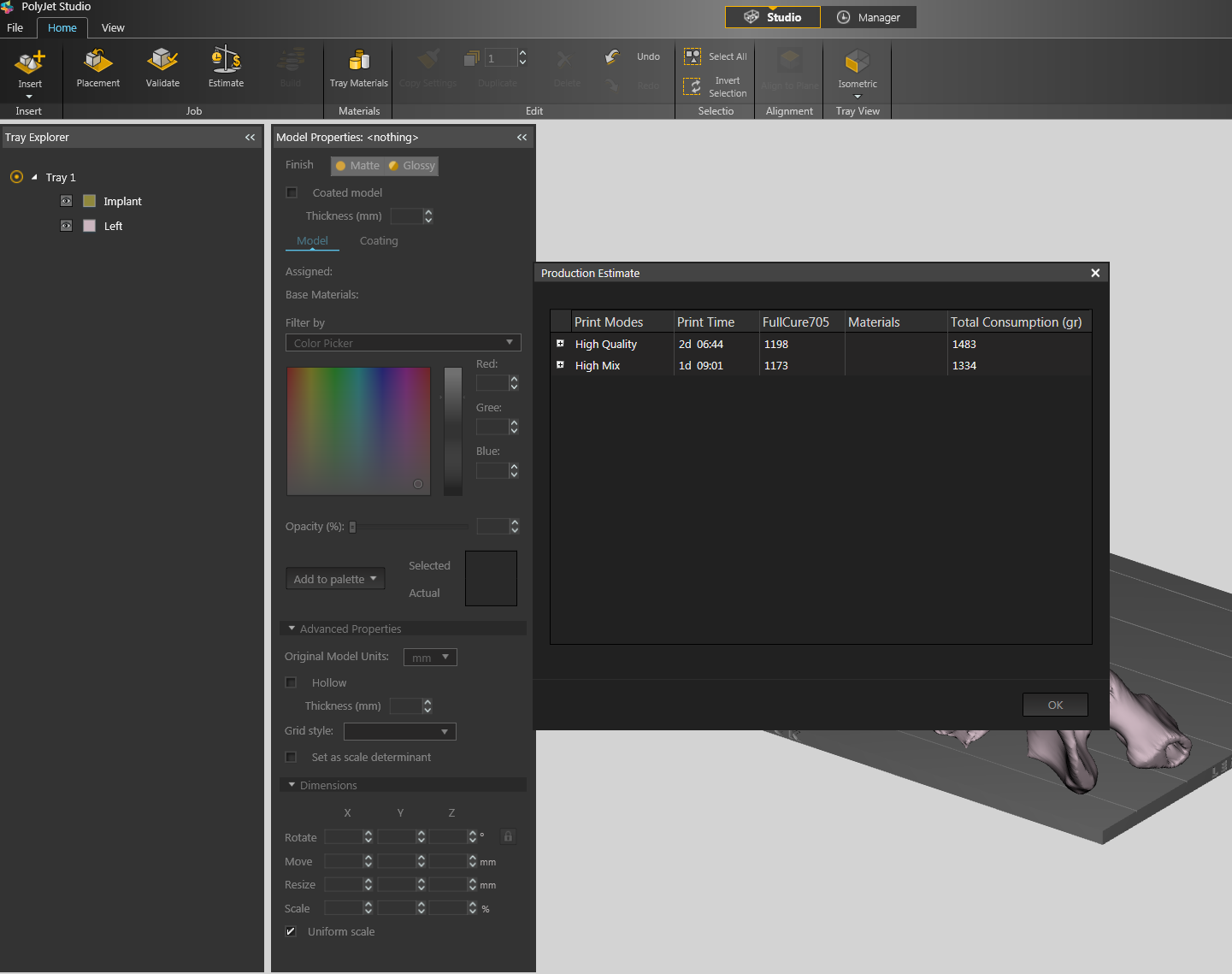


1. In a production environment where the printer is connected to a print server, we would then proceed to the **Manager** tab to connect to the printer, send the job, and ensure that the material store in the printer is sufficient.
