# Supplementary material for: Medical 3D printing for vascular interventions and surgical oncology: a primer for the 2016 radiological society of North America (RSNA) hands-on course in 3D printing
Source: 3D Print Med. 2016 Dec 1;2:5. doi: 10.1186/s41205-016-0008-6 (PMC6036767; doi:10.1186/s41205-016-0008-6)

Appendix A: Creating Holes and Labeling

To create holes in the model, we will again use the **Boolean Subtraction**, but this time, we will need a cylinder to subtract from the model. To do this, simply use the **Design > Create Primitive > Create Cylinder** function. Since we would like to generate two holes, we will create two cylinders. Select **Axis** in the Method section and set up the parameters as below for the first and second cylinders. You can freely translate and rotate any object within 3-matic, allowing you to create cylinders in any position and orientation. However, for illustrative purposes, we will provide all necessary coordinates for the two cylinders here.


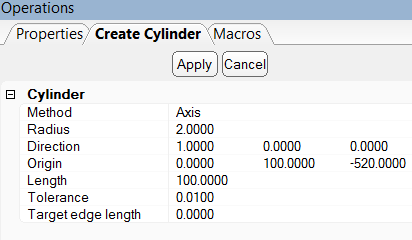

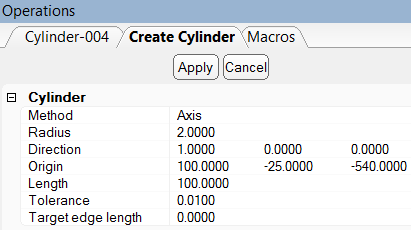


This will produce two cylinders, as shown.


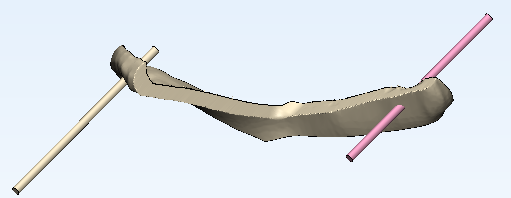


**Boolean Subtraction** can then be used to iteratively remove the two cylinders (which will be subtraction entities), leaving behind 2mm apertures for the positioning pins. Ensure to turn off the **clearance** setting if it is on from prior subtraction.


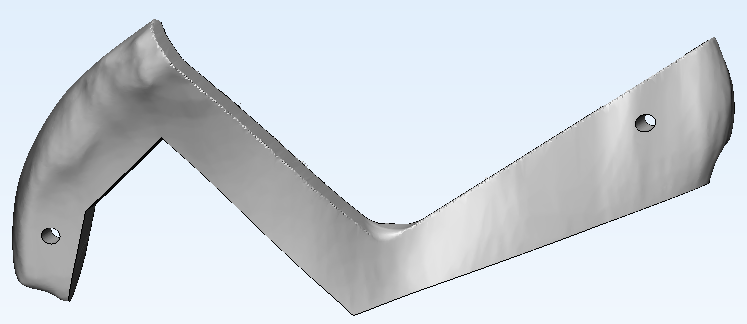


Labeling can be achieved using the **Finish > Quick Label** function. A sample set up for the self-explanatory parameters of this function is shown. The labels produced in the **Text** field are simply placed onto the desired surface of the model by **Left-Clicking** within the area of desired label position.


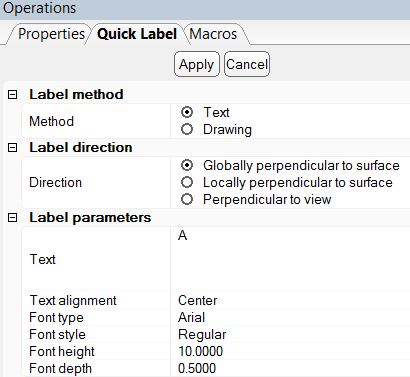


This now results in a properly labelled (A for anterior and P for posterior) surgical guide with holes for positioning pins.


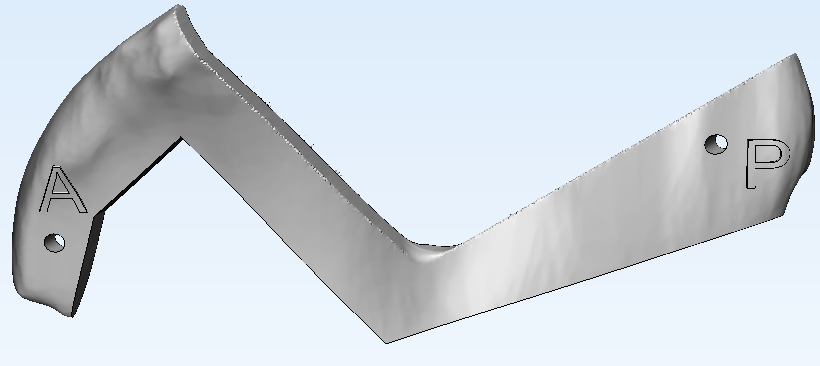

Supplement: Supplementary file 2 — Creating Holes and Labeling. (DOCX 193 kb) [file 41205_2016_8_MOESM2_ESM.docx]
